# Plants and microbes assisted selenium nanoparticles: characterization and application

**DOI:** 10.1186/s12951-014-0028-6

**Published:** 2014-08-16

**Authors:** Azamal Husen, Khwaja Salahuddin Siddiqi

**Affiliations:** 1 Department of Biology, College of Natural and Computational Sciences, University of Gondar, Gondar, Ethiopia; 2 Department of Chemistry, College of Natural and Computational Sciences, University of Gondar, Gondar, Ethiopia

**Keywords:** Nanotechnology, Selenium, Plant extracts, Microbes, Biofabrication

## Abstract

Selenium is an essential trace element and is an essential component of many enzymes without which they become inactive. The Se nanoparticles of varying shape and size may be synthesized from Se salts especially selenite and selenates in presence of reducing agents such as proteins, phenols, alcohols and amines. These biomolecules can be used to reduce Se salts in vitro but the byproducts released in the environment may be hazardous to flora and fauna. In this review, therefore, we analysed in depth, the biogenic synthesis of Se nanoparticles, their characterization and transformation into t- Se, m-Se, Se-nanoballs, Se-nanowires and Se-hollow spheres in an innocuous way preventing the environment from pollution. Their shape, size, FTIR, UV–vis, Raman spectra, SEM, TEM images and XRD pattern have been analysed. The weak forces involved in aggregation and transformation of one nano structure into the other have been carefully resolved.

## Introduction

Selenium was known as a notorious element until it was recognized by Schwarz and Foltz in 1957 as an essential trace element for both plants and mammals. Normally Se is available as selenate and selenite oxoanions. The reduction of soluble Se^4+^ and Se^6+^ by microbes to insoluble non toxic elemental Se is an effective way to remove it from contaminated soil, water and drainage [1]. Se is one of the chalcogens occurring as selenate SeO_4_^2−^, selenite SeO_3_^2−^ and selenide Se^2−^ which may be reduced to atomic state by a precursor containing an appropriate reducing agent. Biogenic synthesis of Se nanoparticles is frequently achieved by reduction of selenate/selenite in presence of bacterial proteins or plant extracts containing phenols, flavonoids amines, alcohols, proteins and aldehydes. The deficiency of Se is known to be associated with over 40 diseases in man [2],[3]. At low dosage it can stimulate the growth of the plant whereas at high dosages it can cause damage to it [4]-[6]. Se has also been shown to be effective against cancer [7],[8]. Their compounds in the form of selenocysteine and selenomethionine are metabolized in biological system [7],[8].

A variety of microorganisms, enzymes and fungi, besides plant extracts have been used to synthesise Se nanoparticles of different size and morphology. Se itself is used in rectifier, solar cells, photocopier and semiconductor. In addition, they exhibit biological activity owing to their interaction with the proteins and other biomolecules present in the bacterial cells and plant extracts, containing functional groups such as ›NH, C = O, COO and C-N [9]. Se-nanobelts have been synthesised on large scale with an approximate diameter of 80 nm and length up to 5 μm [10]. Se exists in many crystalline and amorphous forms but the shape, size and structure of the nanoparticles depend on the concentration, temperature, nature of biomolecules and pH of the reaction mixture. The properties of Se nanoparticles varies with size and shape for instance, Se nanospheres have high biological activity and low toxicity while Se nanowires of t-Se have high photoconductivity [11]. Various methods have been employed to produce large scale Se-nanowires and trigonal selenium (t-Se) [12],[13]. Pulse laser ablation, electro-kinetic technique, hydrothermal treatment, vapour deposition methods [10],[14]-[16] generally used for production of Se nanoparticles on large scale require either sophisticated instruments or specific chemicals which are time consuming and uneconomical. Such methods often employ toxic chemicals or high temperature and high pressure which further pollute the environment.

In order to circumvent the effect of toxic chemicals in the fabrication of nanoparticles, biogenic protocol is generally followed [17],[18]. Scientists have developed benign and harmless methods for the fabrication of nanoparticles using plant extracts, bacteria and fungi. For instance, *Capsicum annum*, *Escherichia coli* and *Bacillus subtilis*[19]-[21] have recently been used to produce nanoparticles. Over 16 different species of bacteria and Arechaea have been found to reduce colourless selenate and selenite to red elemental Se of different shape and size [22],[23]. Plants and microbes act as producers and protectors of the environment when they are properly used. Pure element in its, atomic state may be produced by many bacteria [24],[25] mainly due to the chemicals present in them or protein exuded by them. We have limited knowledge of the mechanism of the formation of Se nanoparticles by microbes and plant extract, nevertheless for a better understanding attempts are being made to explore the chemical reactions occurring in these media. Many bacterial strains have been found to reduce selenate/selenite to Se nanoparticles in different environment [26] even in sewage and sludge under both aerobic and anaerobic conditions [27]-[29]. It has been suggested that substantial quantity of soluble toxic selenate/selenite is reduced by bacterial strain to produce non toxic insoluble Se nanoparticles, although in doing so many such microbes would die. The production of Se^0^/Te^0^ by two anaerobic bacteria *Sulfurospirillum barnesii* and *Bacillus selenireducens* has been demonstrated by Oremland et al. and Baesman et al. [25],[24].

The main objective of this review is to identify the plant extracts and bacterial strains involved in the biosynthesis of Se nanoparticles. Also the characterization and identification of Se-nanoballs, nanorods, nanowires and hollow spheres have been undertaken with a view to update the nanobiotechnology of Se nanoparticles and their application in diverse areas.

### Se nanoparticles from plants, characterization and application

There is a fine line between optimum limit/or deficiency and excess of Se in living system which may cause toxicity. It is known that the Se nanoparticles prepared from biological material are less toxic than the bulk Se nanoparticles prepared from chemicals. The biomolecules present in the extract act both as reducing agent and stabilizers of Se nanoparticles. Bacteria, algae, dry fruits and plant extracts are used to produce nanoparticles. Green synthesis of selenium nanoparticles from selenious acid was achieved by dried extract of raisin (*Vitis vinifera*) [30]. Like other biological materials, raisin also contains sugar, flavonoids and phenols in addition to minerals such as iron, potassium and calcium [19],[31]. A change from colourless to deeply brick-red colour indicated the formation of nanoparticles. The formation of Se-nanoballs was examined at different interval of time. It took nearly 6 min to start conversion of Se ion to Se nanoparticles which was indicated by a decrease in Se ion concentration in the solution. The nature of nanoparticles was analysed by TEM images. It showed that the diameter of nanoballs ranges between 3 and 18 nm. They were found to be encapsulated with a thin polymorphic layer. The formation of Se nanoparticles was confirmed from the energy dispersive x-ray spectroscopy. The Se nanoballs were identified from their characteristic absorption peaks at 1.37KeV, 11.22KeV and 12.49KeV [32]. The morphology of Se nanoparticles can be analysed by x-ray diffraction (XRD) analysis. The broad diffraction peak suggests the presence of amorphous nature of Se nanoparticles [33]. Their particle size [34] has been found to be of the order of 12 nm.

**Figure 1:**
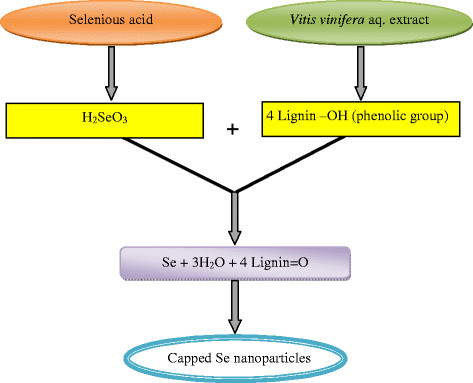
**Se nanoparticle synthesis using** ***Vitis vinifera*** **extract.**

Sharma et al. [30] have characterized Se nanoballs fabricated from *V. vinifera* by FTIR spectral studies. The spectrum exhibited two sharp absorption peaks at 3420 cm^−1^ attributed to OH and, the second peak at 1620 cm^−1^ to C-H vibration of the organic molecules. A distinct peak at 1375 cm^−1^ has been assigned to phenolic OH. The other peaks of medium intensity are due to –CH_3_ and OCH_3_ groups associated with the biopolymers, present in the *V. vinifera* extract acting as reducing agent and stabilizer for the Se nanoballs. Since lignin is a component of all vegetables, fruits and cell wall, it can be extracted from them and the compounds present in them may be identified. In the present work, phenolic group has been identified which generally acts as reducing agent and, it is oxidised to ketone during the redox process. However, the extract also contains fairly substantial amount of reducing sugars and therefore, they also help in the reduction and formation of Se nanoballs. These authors have given a flow diagram for Se nanoparticles synthesis but it does not reveal the chemical changes which occur as a consequence of redox reactions. We now propose the following scheme Figure 1 based on the general synthetic route.

**Figure 2:**
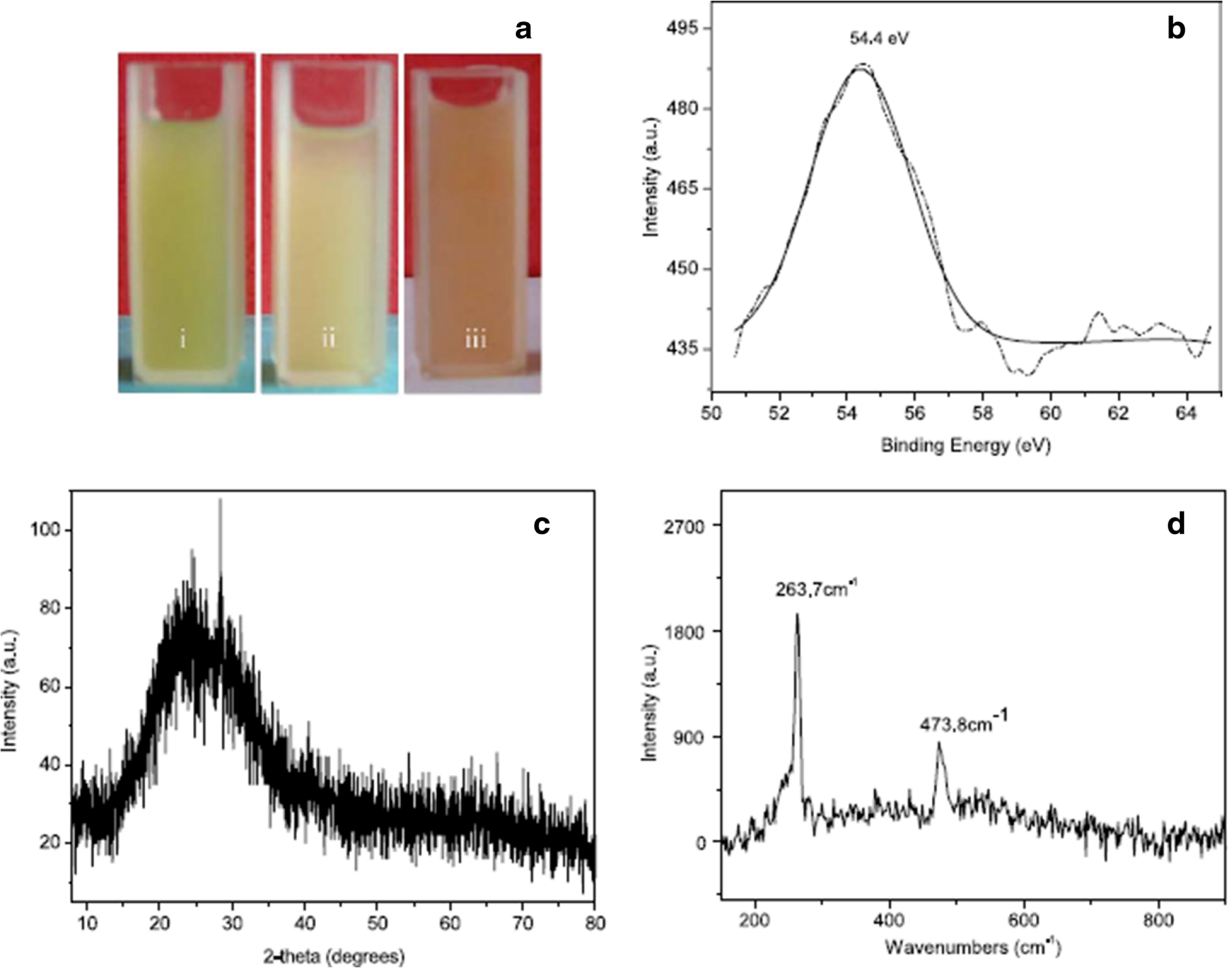
**Se nanoparticle synthesis using*Capsicum annum*extract (a) The time-dependent color changes of the reaction solution; (i), (ii), (iii) represents 0, 5, 15 h, respectively.(b)** XPS spectrum of the product obtained from reaction solution (I). **(c)** Typical XRD pattern of the same product of reaction solution (I). **(d)** Raman spectrum of the same reaction product as in **(c)**[19].

Although, biochemicals may often be used for the synthesis of nanomaterials, the biogenic synthetic route is frequently applied due to its ease and simplicity and, also because no hazardous and toxic residues are released in the environment [35],[36]. In general, a variety of Se nanoparticles are produced when H_2_SeO_3_ is treated with plant extracts for instance, α-Se nanoparticles have been fabricated from *Capsicum annum* extract in aqueous medium at low pH and at ambient temperature [19]. The light green extract of *C. annum* turns pale after 5 h of the addition of H_2_SeO_3_, and then gradually turned red after 12 h (Figure 2a). This red colour is the characteristic signature of α-Se in the x-ray photoemission spectroscopy (XPS) which is due to excitation of their surface plasmon vibration [37]. Its XPS spectrum (Figure 2b) shows a sharp peak at 54.4 eV which corresponds to the elemental selenium [38]. The XRD pattern of the Se nanoparticles shows a broad peak at 2*θ* angles of 15-35^0^ (Figure 2c) which suggests that the nanoparticles are not crystalline. Their Raman spectrum displayed a resonance peak at 263.7 cm^−1^ which (Figure 2d) further confirms the formation of α-Se nanoparticles [39]. An additional peak at 474 cm^−1^ has been attributed to the protein vibration which is mixed with amorphous Se.

SEM and TEM images of the α-Se nanoparticles showed that they consist of nanorods and nanoballs laced with C. annum protein which makes them slightly irregular in shape. The length and diameter of rods and nanoballs range between 1–3 μm and 0.4 μm, respectively. A closer look at the highly magnified field emission scanning electron microscopy (FESEM) image suggests that rod like nanoparticles are actually aggregates of spherical particles with protein coating, making the surface rough and uneven. It is quite likely that proteins are held together by hydrogen bonding and Se nanoparticles are held by van der Waals forces.

When the pH of the reaction mixture is lowered to 2 the time to produce α-Se nanoparticles increases. It has been observed from their FESEM images that a variety of polygonal Se nanoparticles are produced with size varying from 200–500 nm. It is of interest to note that some hollow spherical particles were also produced with a pore diameter of 160 nm. Li et al. [19] have hypothesized that hollow spheres are formed as a consequence of rise in temperature when the reaction product is placed in an electric field. Although, the melting point of α-Se nanoparticles is not very low (~490 K) even then this temperature is seldom achieved in such system, so that it may melt and produce hollow spheres. It is to be noted that even if microwave energy is supplied without rise in temperature only the outer surface of α-Se nanoballs, made of protein layer would melt, because organic materials have inherently lower melting point than metalloid Se. However, if these α-Se particles also melt with electronic impact even then the hollow sphere would not be produced because the lattice would rupture resulting in the formation of irregular sheets and dot like structures. It is more likely, that hollow spheres of α-Se are also formed along with solid nanoballs and polygonal structures during the synthesis of nanoparticles.

A comparison of FTIR spectrum of pure *C. annum* extract with the reaction mixture (*C. annum* extract + SeO_3_^2−^) showed many peaks at 1652, 1542 and 1241 cm^−1^ corresponding to amide I, II and III bands owing to ʋ(C = O), ʋ(N-H) and ʋ(C-N) respectively [36]. These bands slightly shift after the formation of nanoparticles. The UV–vis spectrum of the *C. annum* protein (washed with SDS-PAGE gel) with molecular weight of 30 kDa, showed peak (210 nm) corresponding to peptide bonds and amino residues (280 nm). As these are reducing agents they help in the formation of nanoparticles. It has also been confirmed from cyclic voltammogram that the redox reaction occurs between - 0.7 and 0.9 V [19].

Inorganic Se (selenite or selenate) also occur as selenomethionine, selenocysteine, selenocystathione, methyl selenol, dimethyl selenide and selenium methyl selenocysteine. Absorption of Se depends on its morphology and solubility in aqueous medium. Sodium salts of Se are generally soluble in water. One form may change into another to suit the basic requirements of binding to certain functional groups such as proteins. Different plants absorb Se in different quantities for instance; wheat accumulates Se proportional to its availability in the soil while *Astragalus* grown in the same soil had manifold excess of the element in it. Broccoli is known as Se accumulator. Finley [40], showed from an experiment, on broccoli grown in sodium selenate laden soil, that it accumulated ~ 10^3^ μg Se/gm dry weight of the plant tissue. Broccoli is known to contain fairly [41] large quantity of selenium as methyl selenocysteine, perhaps due to its greater solubility in aqueous medium. However, it is strange that Se from broccoli does not accumulate efficiently in man or rats [42],[43] because its major part as selenium methyl selenocysteine is perhaps metabolised to methyl selenol [44]. It has been demonstrated experimentally that, methyl substituted forms of Se is an effective anticancer agent than the other derivatives of organo-Se compounds [45]. Garlic with as much Se as 1000 μg/gm of dry weight has been grown [46] and found to contain Se as selenium methyl selenocysteine but when the Se concentration falls below 200 μg/gm, it is found as q-glutamyl selenium methyl selenocysteine. Although Se from high Se garlic is a chemoprotective agent [47] it is particularly selective against breast cancer in rats [48],[49] induced by 7, 12-dimethyl benzene (a) anthracene. Even the aqueous solution of garlic is chemoprotective [50]. Glucosinolate as secondary plant compound known to induce phase II enzymes [42] is chemoprotective against bladder cancer. Se fed experimental rats in the forms of selenite, selenate, selenomethionine did not acumulate in most rats which means it is either not absorbed or excreted through urine.

Trigonal Se nanowires and nanotubes have been synthesized in absolutely ecofriendly way. The Se nanowires of 70–100 nm width and length in several μm were prepared in absolute ethanol at room temperature while trigonal Se nanotubes of diameter 180–350 nm were obtained in aqueous medium at 85°C (358 K). It was observed that amorphous Se nanoparticles were formed in the beginning and subsequently transformed into nanowires and tubes [51].

Stable Se nanoparticles in colloidal form were prepared from *Terminalia arjuna* extract in aqueous medium. They were characterized by UV–vis, energy dispersive X-ray analysis (EDAX), transmission electron microscopy (TEM), FTIR and XRD analysis. The colloidal solution had an absorbance maximum at 390 nm. Its IR spectrum showed peaks corresponding to O-H, NH, C = O and C-O stretches suggesting the presence of hydroxyl, amino, ketonic and carbonyl functional groups in the extract which act both as stabilizer and capping agent for the Se nanoparticles [52]. The Se nanoparticles synthesized from fenugreek seed extract in aquous medium at room temperature are between 50–150 nm. They have been found to be active against human breast cancer cells [53].

**Figure 3:**
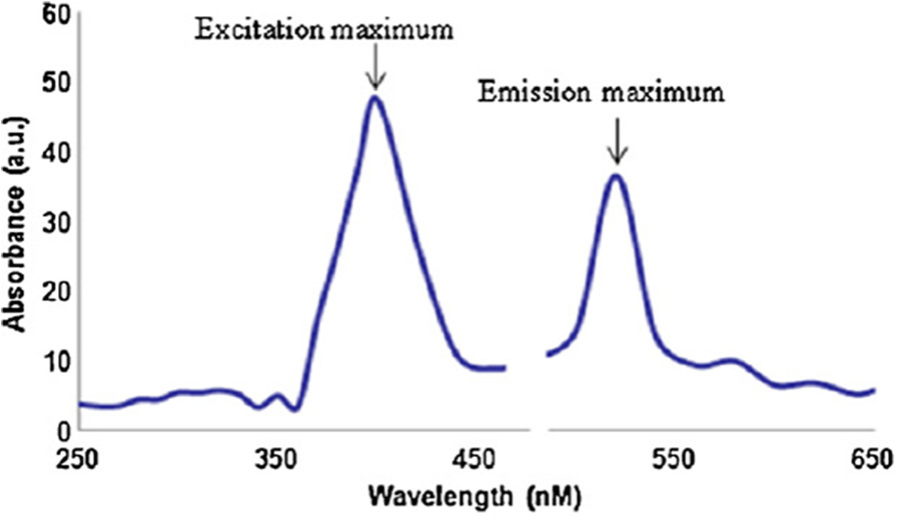
**Photoluminescence spectra of selenium nanoparticles synthesized using leaf extract[**[55]**].**

Se nanoparticles of approximately 35 nm have been synthesized from gum arabic which remain stable in solution for about 30 days. The gum arabic was found to be a better stabilizer for Se nanoparticles than the hydrolysed gum arabic [54]. The Se nanoparticles synthesized from lemon leaf extract exhibited an absorption maximum at 395 nm in the UV–vis region. Initially, the mixture of leaf extract and SeO_3_^2−^ remains colourless but after stirring and incubating it for 24 hr at 30°C, it turns red [55]. The photoluminescence spectra exhibited excitation peak at 395 and emission peak at 525 nm (Figure 3). It has been found from TEM image that the size of particles ranges between 60–80 nm. They are polydispersed in colloidal solution but crystalline in nature [55]. The FTIR spectra of the samples with and without Se nanoparticles were compared to examine the changes in the functional groups of the biomolecules. The broad peak at 3415 due to ʋ(NH) shifts to 3418 cm^−1^ but new peaks appear at 2930 and 3456 cm^−1^ in the colloidal solution containing Se nanoparticles. The region 1500–1800 cm^−1^ is due to various amide bands which split into some new bands in colloidal solution. However, after reduction of the Na_2_SeO_3_ to Se nanoparticles by the biomolecules in the extract containing functional groups such as alcohol, aldehyde, phenol etc., they are oxidized to the following species:

Na_2_SeO_3_ + H_2_O → H_2_SeO_3_ + Na_2_O

H_2_SeO_3_ ⇌ SeO_3_^2−^ + 2H^+^

Alcohol + SeO_3_^2−^ → Se + Carboxylic acid

Aldehyde + SeO_3_^2−^ → Se + Ketone

Phenol + SeO_3_^2−^ → Se + Phenone

It has also been detected from gel electrophoresis that Se nanoparticles prevented DNA damage when cells were exposed to UVB [55].

Polyphenol gallic acid nanoparticles from plant have been used to fabricate Se nanoparticles since the gallic acid nanoparticles may behave differently than the bulk gallic aicd. Since gallic acid can quantitatively coordinate with the Se ions, another reducing agent dithioerthreitol was added to gallic acid-coated with Se ions. A change in colour was taken as an indication of the formation of nanoparticles which was confirmed by UV–vis and emission spectroscopy [56]. A slightly different method has also been employed by Ingole et al. [34] to prepare Se nanoparticles from glucose. Na_2_SeSO_3_ prepared from Se powder was treated with glucose solution according to the following chemical reactions:

Se powder + Na_2_SO_3_ → Na_2_SeSO_3_

Na_2_SeSO_3_ + H_2_O → H_2_SeO_4_ + Na_2_S

H_2_SeO_4_ ⇌ 2H^+^+SeO_4_^2−^

SeO_4_^2−^ + Glucose → Se + Gluconic acid

The colourless solution in the beginning becomes yellow then orange and finally turns red which does not change even after heating for over 1 h. These changes have been ascribed to the changes in size of Se nanoparticles.

### Se nanoparticles from microbes, characterization and application

Microorganisms reduce the toxic, selenate and selenite oxoanions into non toxic elemental selenium which is insoluble in water. Continuous use of water or edible plants from Se rich soil can cause skin lesion and early hair fall. Effort is therefore, made to reduce Se compounds to elemental Se with the help of bacteria. It is a simple process of detoxification of selenites/selenates to Se nanoparticles as the reverse reaction is too slow to produce Se compounds [57].

Fast (forward reaction)

Se(IV)/Se (VI) ⇌ Se(o)

Slow (backward reaction)

**Figure 4:**
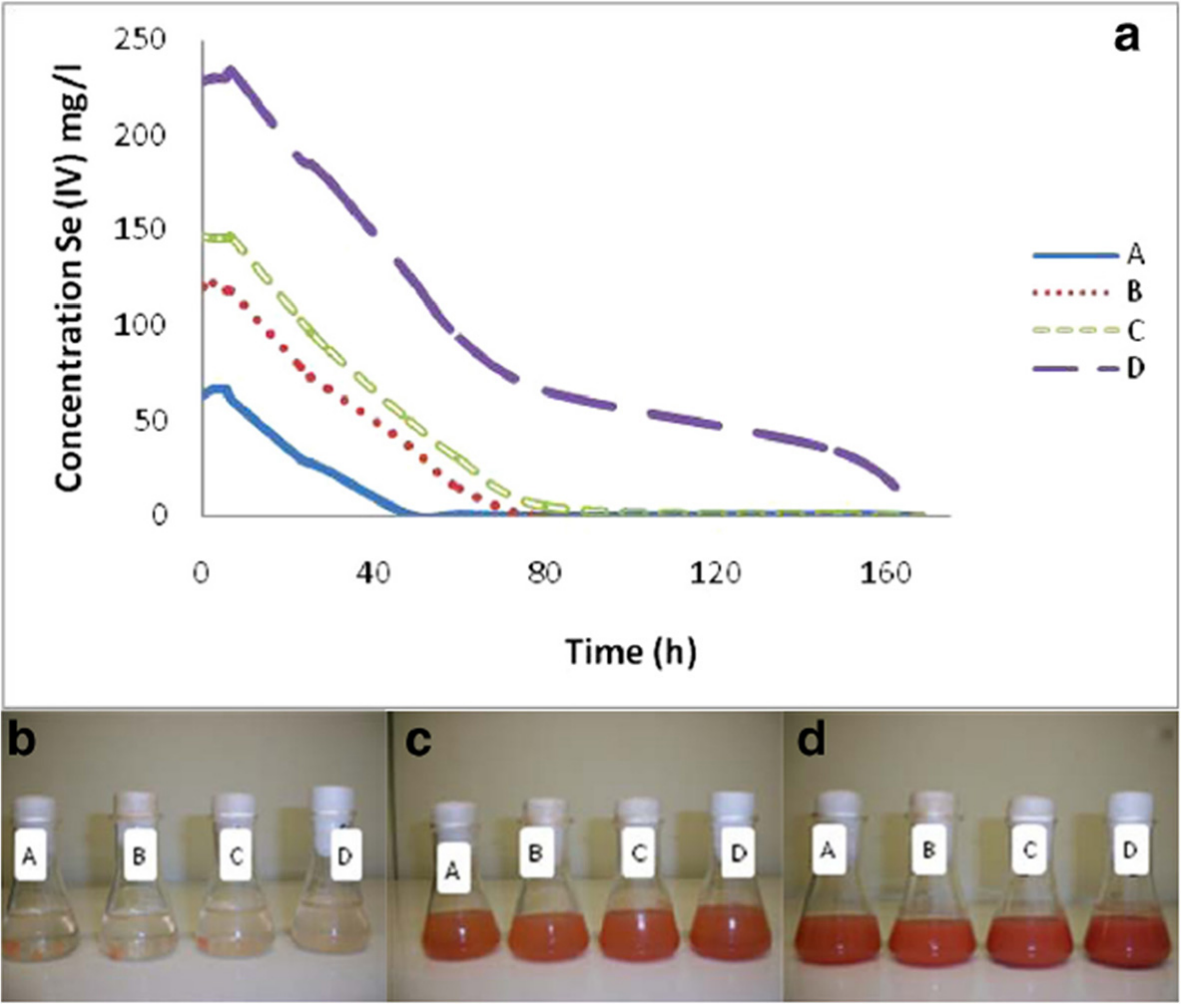
**Selenite reduction by the mixed microbial culture isolated from agricultural soil.** Selenite reduction at different Se (IV) concentrations **(a)** and development of red coloration in cultures after 5.5 h **(b)**, 23 h **(c)** and 48 h **(d)** of incubation [58].

Due to their unique property Se nanoparticles are photovoltaic and semiconductor, antioxidant and chemoprotective agents [58]. Since Se nanoparticles inhibit the growth of *Staphylococcus aureus* it can be used as a medicine against *S. aureus* infection. Different concentration of Se starting from 65 to 230 mg/L of Se(IV) were allowed to interact with different types of microbes. Appearance of red colour was taken as sign of reduction of Se(IV) to Se(0) as shown in forward reaction above. However, there was no decolouration later, indicating the absence of any species causing oxidation of Se(IV) → Se(VI) (Figure 4). The redox process is time and concentration dependent. When bacterial culture was grown in presence of 40–100 mg/L selanate, no change in colour was observed even after long time. It appears as if the bacteria are resistant to Se(VI) reduction. However, such bacterial culture may be used to reduce soluble and toxic Se(IV) to non toxic and insoluble Se nanoparticles. It is also indicative of bioremediation of Se from selenites.

Amporphous Se nanoparticles have been synthesized from sodium selenite in presence of *Shewanella* sp. HN-41 in aqueous medium under anaerobic conditions taking care of reaction time, selenite concentration and biomass of *Shewanella*[59]. Different types of the Se nanoparticles are synthesized using protein, peptides and several other reducing agents [1],[60],[61]. Nanowires and nanorods have been fabricated from *Rhizobium selenireducens* sp., *Dechlorosoma* sp., *Pseudomonas* sp., *Paracoccus* sp., *Enterobactor* sp., *Thaurea* sp., *Sulfurospirillium* sp., *Desulfovibrio* sp., and *Shewanella* sp., [61]-[63]. It has been reported that the particle size is decreased in presence of O_2_. It is obvious that oxygen will promote oxidation of Se (forward reaction) as a consequence of which the redox step becomes slower producing smaller Se nanoparticles. Selenite reductase is also helpful in the synthesis of Se nanoparticles. A wide range of selenite concentration starting from 0.01, 0.05, 0.15, 0.25, 0.050, 0.75 and 1 mM were used to study the effect of concentration, size and morphology. Average particle size for all the above concentrations was nearly 103 ± 5.1 nm. For large quantity of nanoparticles the selenite concentration not exceeding 0.1 mM is needed. It has been observed from TEM and SAED image that Se nanoparticles are amorphous [59]. Extracellular synthesis [64] of fairly smaller particles of the order of 47 nm from the fungus, *Aspergillus terreus* was done in 60 min.

Microbes like *Klebsiella pneumoniae*[31] and *Pseudomonas alcaliphila*[65] have also been used to synthesize Se nanoparticles in good yield. When Na_2_SeO_3_ was added to the activated culture of *P. alcaliphila* the reaction started immediately but completed after 48 h [65]. A gradual colour change with time was observed in the following order:

Grey → Red → Intense Red

0 h 6 h 48 h

The characteristic red colour of Se nanoparticles [37] was detected spectrophotometrically and has been ascribed to the excitation of the surface plasmon vibration of the monoclinic Se. It has been noticed that particle size is directly proportional to reaction time and it ranges between 50–500 nm [21]. Field emission scanning electron microscopic (FESEM) image shows nanoparticles of varying size and shape.

**Figure 5:**
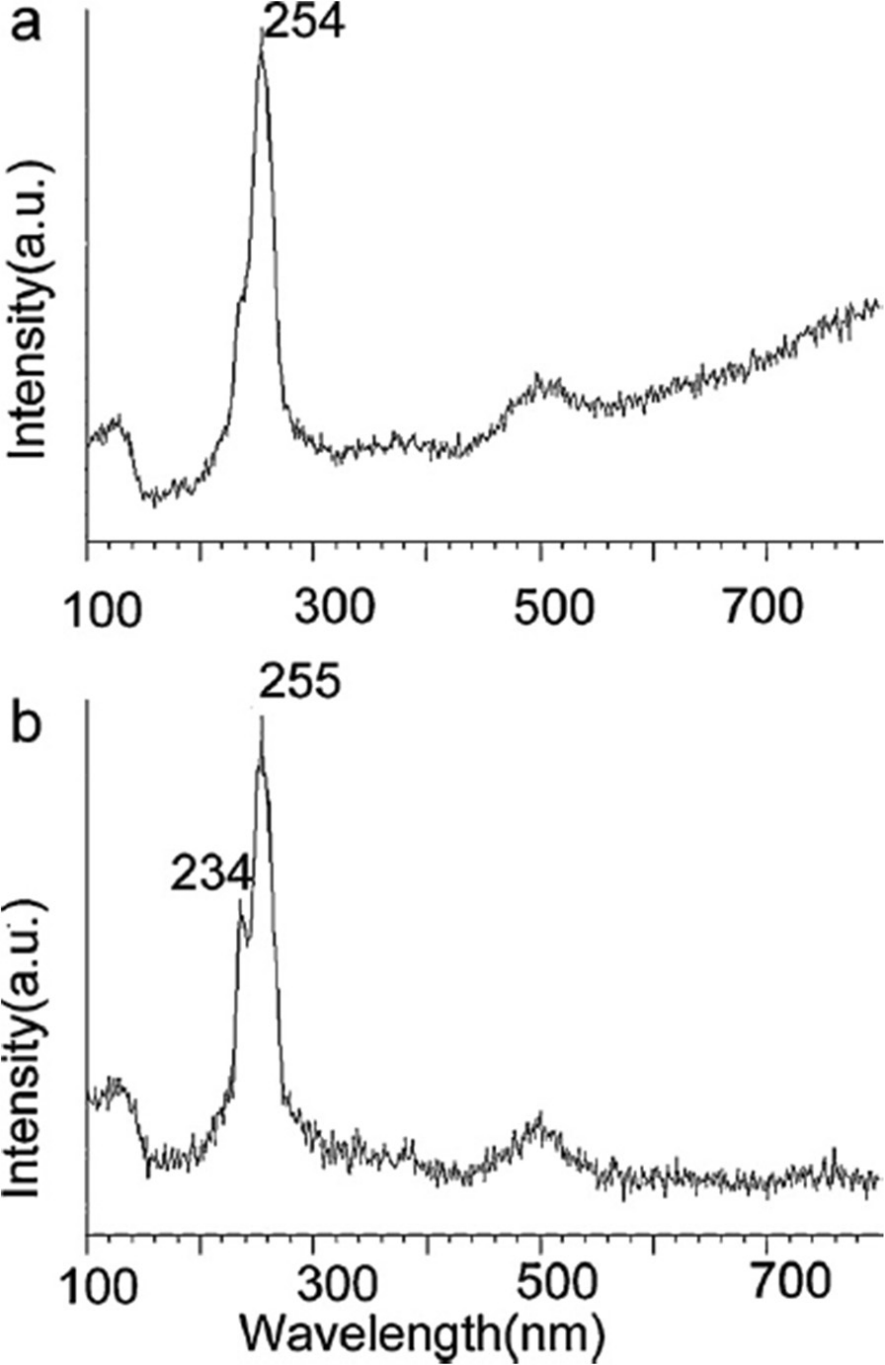
**Raman scattering spectra of SeNPs trapped at different incubation times: (a) 24 h and (b) 48 h[**[65]**].**

The FTIR spectra of the samples with and without Se nanoparticles showed that the intensity of the spectral peaks containing Se nanoparticles is drastically diminished [65] which suggests strong interaction between Se atoms and the protein molecules present in the *P. alcaliphila*. This is to be noted that the interaction between Se nanoparticles and protein is simply electrostatic because the intensity of sample containing Se atoms was decreased followed by an increase in ʋ(NH) from 3421 to 3435 cm^−1^. The Raman spectra also support the formation of trigonal selenium (t-Se) and monoclinic selenium (m-Se) by the appearance of peaks at 234 and 254 cm^−1^, respectively (Figure 5). A peak at 235 cm^−1^ is mainly due to chain like structure of t-Se. As the peak at 234 cm^−1^ appears after 48 h of inoculation, it is considered as the transformation of one form of Se into other. The FESEM images which show the accumulation of nanorods on the nanoballs. The size can be controlled by PVP at different time of incubation of nanospheres ranging from 20–200 nm.

On the basis of above studies a possible mechanism for the formation and transformation of Se nanoballs to Se nanorods has been proposed. Zhang et al. [65] presume that SeO_3_^2−^ is reduced to elemental selenium by the protein excreted by the *P. alcaliphila* and, their aggregation gives Se nanoparticles of varying size [13]. It is true that protein acts as reducing agent for SeO_3_^2−^ but it is available as excretion from *P. alcaliphila* is not convincing. The excretion contains toxins, pyrogens and traces of protein but they may not be sufficient for reduction of selenite. Authors also suggest that large m-Se nanoballs are not stable in solution and they dissolve to form Se atoms. A fraction of dissolved Se atoms crystallize as t-Se forming nanorods [66]. It is not rue because Se in atomic state is not soluble in a solvent but stays in colloidal form. It is a misconception. However, PVP controls the size of Se nanoparticles. If the Se nanoparticles without PVP are left for 2–3 weeks they form aggregates of different shapes and size. Since SeO_3_^2−^ in ionic form is toxic to bacterial culture, the bacteria in selenite solution may therefore, die and the disintegrated protein may then act as reducing agent for selenite. The large m-Se nanoparticle can not dissolve in solution to give Se atom forming Se-nanorods. It is quite likely that they may be segregated and rearranged into nanorods.

Se nanoparticles synthesized from sodium selenite and glutathione in aqueous medium had been tested for its growth inhibition efficacy against *Staphylococcus aureus*[67]*. It was found that growth of S. aureus was inhibited in presence of Se nanoparticles within 3–4 h with as low concentration as 7–15 μg/ml which suggests that Se nanoparticles may be used against bacterial infections.*

Biogenic Se nanoparticles fabricated from protein produced by *E. coli* have been compared with those synthesized from chemical reaction via redox mechanism [68]. There are specific types of protein produced by *E. coli* (AdhP, Idh, OmpC, AceA) which are associated with Se nanoparticles. They are also responsible for their uniform size and distribution. One of the proteins (AdhP) was found to bind strongly to Se nanoparticles. *E. coli* was found to produce Se nanoparticles from 2 mM of SeO_3_^2−^ in about 48 h which was distinguished by the change in colour from colourless to dark red. The amorphous Se nanoparticles thus produced were between 10–90 nm. Since the bacterial growth continued even in presence of selenite, it is confirmed that selenite is not toxic to *E. coli* at this concentration. The enzymes alcohol dehydrogenase, propanol-preferring (ADHP), ACEP (Isocitrate lyase), ENO, KPYKI, IDH and GLPK require metal ions as cofactor for their activity while the enzymes DCEP, ASTC and TNAA require pyridoxal phosphate as coenzyme without which their activity is lost. The authors have not distinguished between cofactor and co-enzyme, they have termed both as metallic cofactor and non-metallic cofactor which is misleading. The cofactors in the enzyme are metal ions bonded through a coordinate covalent bond and can accept lone pair of electrons from the donor atoms in the enzyme into their vacant orbital to form the bond. The Se nanoparticle is in the elemental state and no metal in atomic state can bind to protein or any electron donating species. It is therefore; proper to use the word association of Se nanoparticles to protein rather than bonding. The size of Se nanoparticles produced in presence of the protein, alcohol dehydrogenase propanol-preferring (AdhP) were much smaller than those produced in their absence. However, since *E. coli* contains many other proteins than only pAdhP (purified protein), the decrease in Se nanoparticle size may be the cumulative effect of all proteins available in *E. coli*.

## Conclusion

Bioreduction of selenate or selenite from microorganism such as bacteria, fungi and plant extract have become the favourite pursuit of biologist, chemist and engineers. It is expected that in future the metal would be extracted by biomineralization because they produce the purest form of the element. Many raw materials like waste vegetables, fruit peels and leather cuttings may be utilized to produce elemental metal/metalloid from their oxide, halide, nitrate, sulphide and carbonates. Generally, protein, phenol, alcohol, flavonoid or sugar are required for the reduction of SeO_3_^−2^, SeO_4_^−2^ and at least one of the above organic molecules is present in microbes and plant extracts. They may therefore, be exploited for the biotransformation of selenate and selenide to elemental Se of various shape and size. Since the reduced metals or metalloids are insoluble in aqueous medium they can be easily sequestered. Growth inhibition of some of the bacterial stains occurs during the redox process which suggests that selenite/selenate may be used against infection caused by such microbes.

## Competing interests

The authors declare that they have no competing interests.

## Authors’ contributions

AH gathered the research data. AH and KSS analysed these data findings and wrote this review paper. Both authors read and approved the final manuscript.
